# Nephrotoxicity Associated with Cytoreductive Surgery Combined with Cisplatin-Based Hyperthermic Intraperitoneal Chemotherapy for Peritoneal Malignant Disease: A Systematic Review and Meta-Analysis

**DOI:** 10.3390/jcm13133793

**Published:** 2024-06-28

**Authors:** Cristián Grillo-Marín, Cristina Antón-Rodríguez, Lola Prieto, Gloria Ortega-Pérez, Santiago González-Moreno

**Affiliations:** 1 Department of General Surgery, Hospital Universitario Puerta de Hierro, Calle Joaquín Rodrigo 1, 28222 Majadahonda, Spain; cgrillomarin@gmail.com; 2 Unidad de Apoyo a la Investigación, Facultad de Medicina, Universidad Francisco de Vitoria M-515, km 1, 800, 28223 Pozuelo de Alarcón, Spain; c.anton@ufv.es (C.A.-R.); lola.prieto@ufv.es (L.P.); 3 MD Anderson Cancer Center, Madrid Spain Foundation, Calle Arturo Soria 270, 28033 Madrid, Spain; 4 Department of Surgical Oncology, Peritoneal Surface Oncology Program, MD Anderson Cancer Center, Calle Arturo Soria 270, 28033 Madrid, Spain; gortega@mdanderson.es

**Keywords:** cisplatin, hyperthermic intraperitoneal chemotherapy, kidney injury, meta-analysis, nephrotoxicity

## Abstract

**Background:** Cisplatin is employed in hyperthermic intraperitoneal chemotherapy (HIPEC) after cytoreductive surgery (CRS) for peritoneal surface malignancies (PSMs). The main concern regarding intraperitoneal cisplatin administration is nephrotoxicity. Numerous reports in this context are available. Our objective was to conduct a systematic review and meta-analysis to assess cisplatin-based HIPEC-related nephrotoxicity (CHRN). **Methods:** A systematic literature review on CHRN after CRS for the treatment of PSMs was performed. The literature search was carried out using Medline, Cochrane, and Embase. The last day of the search was 23 October 2023. PRISMA guidelines were used. A meta-analysis was then conducted. The main endpoint was the incidence of acute and chronic renal impairment after CHRN. Secondary endpoints included the potential impact of several clinical variables on the primary endpoint and a critical appraisal of the different renal impairment scales employed. **Results:** Our study included 26 articles with a total sample of 1473 patients. The incidence of acute kidney injury (AKI) was 18.6% (95% CI: 13.6–25%, range of true effects 3–59%). For chronic kidney disease, it was 7% (95% CI: 3–15.3%, range of true effects 1–53%). The variables that statistically influenced these results were the scale used to measure renal insufficiency, the use of nephroprotective agents, and the presence of pre-existing renal disease. **Conclusions:** The reported incidence of renal impairment following cisplatin-based HIPEC is highly variable. The incidence of renal failure obtained in this meta-analysis should be used as a reference for subsequent reports on this topic. Further prospective studies are warranted to establish optimal and standardized management.

## 1. Introduction

Peritoneal surface malignancies (PSMs) are one of the most significant clinical challenges in modern oncology. Ovarian cancer ranks as the third most prevalent gynecological [1] malignancy in women, with approximately 75% of cases being diagnosed at advanced stages (IIIB–IV), which feature peritoneal metastases (PMs) [2]. In gastric cancer patients [3], PMs are present in 5–20% of cases eligible for potentially curative resection. PMs from colorectal origin are common, affecting 10–30% [4] of patients overall and approximately 7–8% at the time of primary surgery.

During the last decades, the use of hyperthermic intraperitoneal chemotherapy (HIPEC) after complete cytoreductive surgery (CRS) has become an effective therapeutical strategy for peritoneal surface malignancies. Cisplatin is among the chemotherapeutic drugs most commonly employed in HIPEC, either alone or in combination with other agents (i.e., mitomycin C, doxorubicin). It shows one of the best profiles for this use given its depth of penetration in the tumor nodules ranging from 1 to 5 mm [5] and its cytotoxic synergy with hyperthermia.

Cisplatin [6] (cis-diaminodicloroplatino(II), CDDP) is an alkylating agent widely used for the treatment of different tumors. It has demonstrated effectiveness in neoplasms of gastrointestinal and gynecological origin. CDDP administration may cause ototoxicity, neurotoxicity, or vomiting. However, its main concerning side-effect is nephrotoxicity, which may appear either after systemic or intraperitoneal administration. The incidence of renal injury following the intraperitoneal administration of CDDP exhibits considerable variability, with reported rates [7] ranging from 3% to 48% across different series. CDDP is known to cause proximal renal tubular damage resulting in subsequent acute or subacute tubular necrosis which may progress to acute renal failure requiring temporary dialysis or, furthermore, chronic renal failure. Different agents are being used to prevent cisplatin-induced nephrotoxicity secondary to HIPEC with CDDP, like sodium thiosulfate, cilastatin or amifostine, but no definitive evidence or standardized protocols about their use has been established.

Two pivotal phase III trials emerged in 2023 establishing the role of HIPEC with CDDP for PMs from epithelial ovarian cancer in two specific scenarios: interval CRS after neoadjuvant therapy (OVHIPEC-1 [8]) and platinum-sensitive peritoneal relapse (CHIPOR [9]). The ten-year [10] follow-up results from OVHIPEC-1 confirmed the overall and disease-free survival benefit of HIPEC with CDDP in FIGO stage III ovarian cancer after CRS. Furthermore, the OVHIPEC-2 trial is currently studying its role in upfront CRS for this disease. Several studies are pending publication with promising results on various cancer types. Conversely, a significant rise in the adoption of this treatment strategy in regular clinical practice is to be expected in the near- to mid-term future.

Although CRS and HIPEC are curative-intent oncological therapeutic approaches, patients usually require additional systemic chemotherapy. Consequently, the preservation of an optimal renal function is essential for comprehensive patient care. The actual incidence of cisplatin-induced nephrotoxicity after HIPEC has not been completely established, since its figures and the renal impairment scales used in different publications are highly heterogeneous. Understanding its safety range in a context of its expected rise in clinical practice use is fundamental in order to standardize safe treatment protocols.

## 2. Materials and Methods

We performed a systematic literature review and meta-analysis to determine the incidence of CDDP-based HIPEC-induced renal toxicity. Secondary aims were to explore different nephroprotective practices used in this context and the assessment of the influence of different clinical variables on reported nephrotoxicity, including the variability of the renal impairment scales employed.

The study protocol was submitted to the PROSPERO [11] database (registered as CRD42021251338). This systematic review is compliant with the Preferred Reporting Items for Systematic Reviews and Meta-Analyses (PRISMA) guidelines [12].

### 2.1. Data Collection

The literature search was performed using the MEDLINE, Cochrane, and EMBASE databases. The last search was performed on 23 October 2023. The following free-text terms and Medical Subject Headings (MeSH) and their combinations were used: “hyperthermic intraperitoneal chemotherapy”, “hyperthermic”, “intraperitoneal”, “chemotherapy”, and “kidney”.

### 2.2. Eligibility Criteria

The included studies are observational studies (prospective or retrospective), along with randomized and non-randomized clinical trials involving more than 10 participants aged 18 years or older. Enrolled patients must have a diagnosis of peritoneal malignant disease, either primary or metastatic, treated with cytoreductive surgery and cisplatin-based HIPEC. Those treated with a combination of cisplatin and doxorubicin were admitted due to doxorubicin’s [13] toxicity profile, which does not include renal impairment, contrary to mitomycin C [14]. Therefore, for the purpose of this paper, the term “cisplatin-based HIPEC” includes cisplatin alone and combinations of cisplatin and doxorubicin. Letters, case reports, editorials, cohorts with fewer than 10 patients, studies without full-text article availability, and studies written in non-English languages were excluded.

Reported outcomes had to include information regarding and incidence of renal impairment according to an objective renal impairment scale. These included the Kidney Disease Improving Global Outcomes (KDIGO) [15], Acute Kidney Injury Network (AKIN) [16], Risk, Injury, Failure, Loss, and End-Stage (RIFLE) [16], Clavien–Dindo [17], or National Cancer Institute Common Toxicity Criteria for Adverse Effects (CTCAE) [18] version 5.0 classifications.

### 2.3. Study Selection and Data Extraction

All the identified studies were peer-reviewed in couples after deleting duplicates. Firstly, double-blind title and abstract screening was performed (CA and LP). Subsequently, full-text study assessment was carried out identifying which studies fulfilled all the inclusion criteria (CG and SG). Dissensions were resolved by a third reviewer (GO). This procedure was performed using Rayyan Legacy [19] free software. Later, data extraction including the variables listed in Table 1 was performed (CG).

### 2.4. Outcome Measures

The primary endpoint of this systematic review and meta-analysis is the incidence of renal impairment, including both acute and chronic disfunction. To assess secondary outcomes, i.e., various clinical variables that may influence the incidence of renal impairment following HIPEC, several subgroup analyses were conducted. The following clusters were investigated: nephroprotective strategies, chemotherapeutic agents employed for HIPEC, dosage of cisplatin administered, renal impairment grading scale employed, etiology of PSM, pre-existing renal conditions, gender and age distribution, as well as study design.

Some of the included studies directly compare, in two arms, the use or non-use of pharmacological nephroprotection strategies. In order to evaluate whether this intervention influences the incidence of renal impairment, a specific interventional comparison meta-analysis was conducted in addition to the overall study.

### 2.5. Statistical Analysis

After data extraction, a comprehensive study of the incidence of acute renal failure following CRS and cisplatin-based HIPEC was conducted, followed by a subgroup analysis, as described earlier. A one-arm statistical analysis was conducted using Comprehensive Meta-Analysis Version 4 software [20]. Revman 5 [21] was utilized for two-arm meta-analysis to evaluate the relative risk of AKI with or without nephroprotection.

The random-effects model was employed for the analysis [22], under the assumption that the studies included represent a random sample from a broader universe of potential studies, and that the results will be extrapolated to that universe. Significance was established at a 95% confidence interval (CI), with *p* < 0.05 considered statistically significant. Heterogeneity analysis was performed using the I-squared (I^2^) statistic, which provides a percentage indicating the proportion of variance in the observed effects attributable to true effects rather than sampling errors. The overall analysis also includes the predictive interval, in which the incidence of all the 95% comparable population falls assuming that true effects are normally distributed.

### 2.6. Methodological Quality of the Included Studies and Risk of Bias Assessment

Following the Cochrane recommendations, the Joanna Briggs Institute (JBI) Critical Appraisal tool for use in systematic reviews of randomized [23] and non-randomized clinical trials [24], case series [25], cohort studies, and case-control studies [26] was used for methodological quality (risk of bias) study assessment. Each study was assessed twice based on the risk classification criteria. Studies with positive answers ≤49% were considered to have a high risk of bias; studies with scores between 50–69% were classified with a moderate risk of bias; and those with scores above 70% were categorized as studies with a low risk of bias.

## 3. Results

A total of 572 records were found. After deleting duplicates, 472 records remained for assessment. First screening by title and abstract excluded 352 records, leaving 120 articles for full-text assessment. Finally, 27 studies were included for qualitive synthesis and 26 for quantitative synthesis. The reason why one article [27] was excluded for quantitative synthesis is that the data reported were aggregated and therefore could not be discretely analyzed.

**Figure 1:**
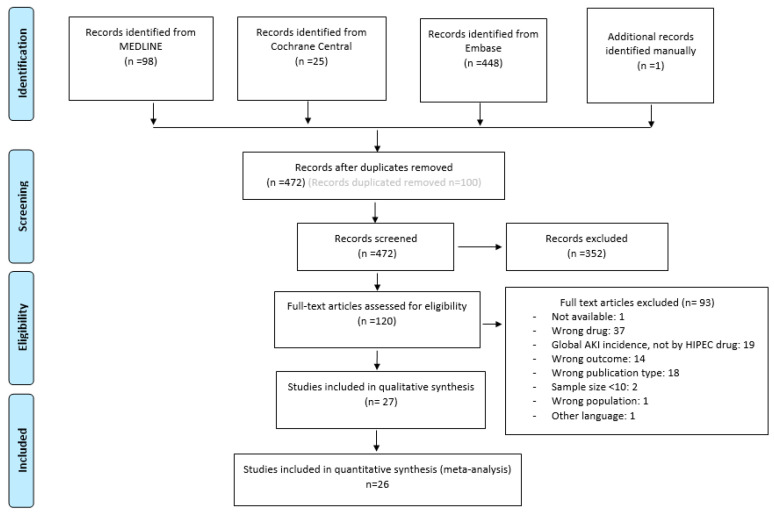
PRISMA diagram [12].

The reasons for the exclusion of 93 studies during the full-text assessment for eligibility along with the overall study selection process are shown in the PRISMA diagram (Figure 1), for more detail see Supplementary Material [7,10,28,29,30,31,32,33,34,35,36,37,38,39,40,41,42,43,44,45,46,47,48,49,50,51,52,53,54,55,56,57,58,59,60,61,62,63,64,65,66,67,68,69,70,71,72,73,74,75,76,77,78,79,80,81,82,83,84,85,86,87,88,89,90,91,92,93,94,95,96,97,98,99,100,101,102,103,104,105,106,107,108,109,110,111,112,113,114,115,116,117].

### 3.1. Characteristics of Included Studies

There were eleven cohort studies, one cases series, two case-control studies, five non-randomized clinical trials, and one randomized clinical trial (Table 2) Regarding the chemotherapeutic regimens utilized during HIPEC, four studies employed a combination of cisplatin and doxorubicin, twenty used cisplatin alone, and two studies used both in different arms (Liesenfeld et al. [117] and Somashekhar et al. [118]). Regarding nephroprotective strategies, four studies employed sodium thiosulfate and two used amifostine. In four studies (Lim et al. [119]; Laplace et al. [6].; Senguttuvan et al. [120]; and Glennon et al. [121]), the incidence of renal failure following the intervention is directly compared in two separate arms. Various criteria for renal impairment assessment were utilized: RIFLE was employed in six instances, WHO and AKIN were each used once, CTCAE nine times, Clavien–Dindo in three studies, and KDIGO four times.

### 3.2. Assessment of Risk of Bias

The overall risk of bias in the analyzed studies was low, showing a high percentage of positive answers to the JBI Critical Appraisal Tools assessment (Table 3A–D). Three non-randomized clinical trials had a moderate risk of bias (Table 3E). In these studies, negative evaluations were associated with the lack of a control group. Likewise, unclear responses in studies with moderate bias were linked to the lack of information regarding appropriate group comparisons.

### 3.3. Quantitative Synthesis

After analyzing the 26 studies included in the meta-analysis, a total of 1473 patients who underwent CRS followed by HIPEC based on cisplatin, as defined above, were available for analysis.

#### Primary Endpoint: Incidence of Acute and Chronic Renal Impairment

**Figure 2:**
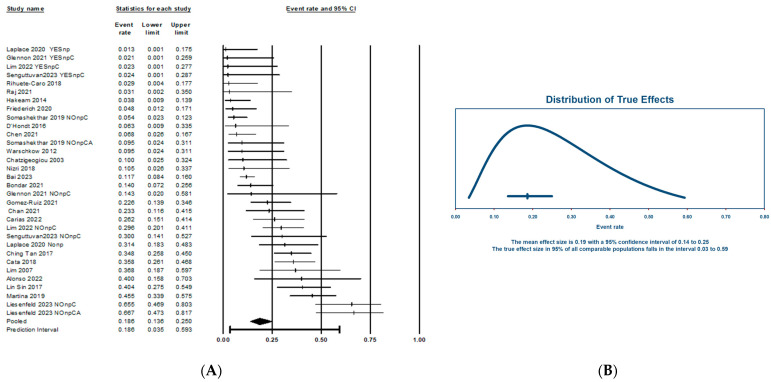
(**A**) One-arm forest plot of acute kidney injury (AKI) incidence after the use of HIPEC in carcinomatosis patients. (**B**) AKI incidence true effects distribution (predictive interval).

The obtained results reflect an overall acute kidney injury (AKI, Figure 2A) incidence of 18.6% (95% CI: 13.6–25%). The observed high heterogeneity (I^2^ = 82) is reflected in a wide range of true effects, producing an expanded predictive interval of 3–59%. (Figure 2B). This suggests that, by eliminating potential sources of error and bias through mathematical modeling, the incidence of AKI following cisplatin-based HIPEC would fall within the range of 3% to 59%. The I-squared (I*^2^*) statistic reflects that 82% of the variance in the observed effects reflects variance in true effects rather than sampling error.

**Figure 3:**
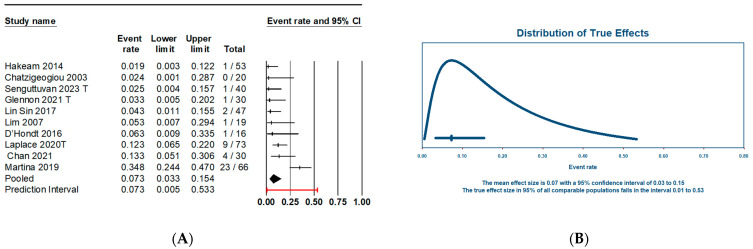
(**A**) Forest plot of chronic kidney disease (CKD) incidence after the use of HIPEC in PSM patients. (**B**) CKD incidence true effects distribution (predictive interval).

Another primary endpoint of this meta-analysis is the estimation of the global incidence of chronic kidney disease (CKD) following the intervention (Figure 3A). The results reveal a CKD rate of 7% (95% CI: 3–15.3%). Similarly to the AKI rate, the observed heterogeneity is high (I^2^ = 75.89%). When calculating the range of true effects (Figure 3B), the CKD incidence would fall between 1–53%.

Given the high heterogeneity and the wide renal impairment range, the incidence rate has been disaggregated based on variables considered of interest for its possible impact on the observed effect. These variables (Table 1) are HIPEC regimen, cisplatin dose, HIPEC duration, etiology of PSM, incidence of acute renal injury with or without nephroprotection (single-arm or double-arm comparative studies), pre-existing chronic renal disease, and scale of renal impairment used. To carry out this analysis, some variables have been categorized. The breakdown analysis based on the previously mentioned variables is shown in Table 4. Among them, two yield statically significant results: the scale used to measure AKI and previous chronic kidney disease.

**Figure 4:**
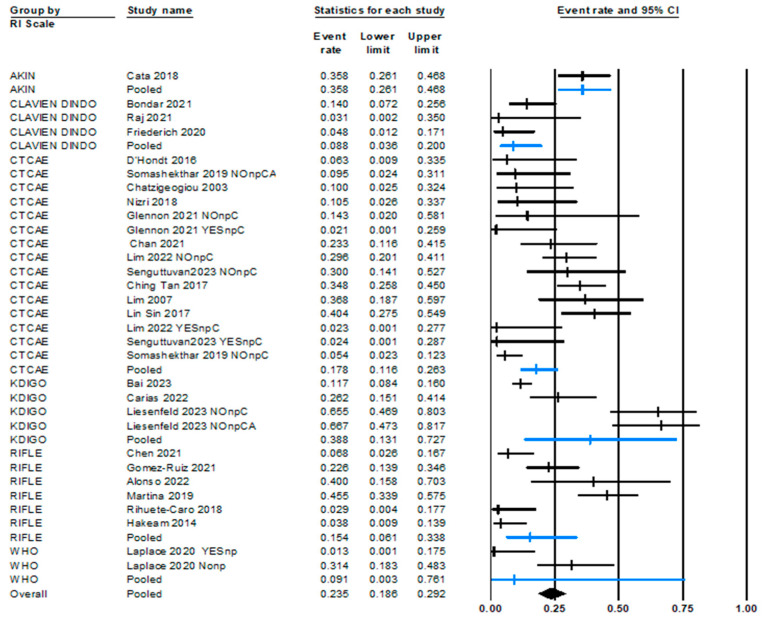
Forest plot of AKI incidence depending on the scale used.

Regarding the use of renal insufficiency scales, the overall results (Table 4) obtained show that there are significant differences between the AKI incidences among the scales employed for assessing renal failure. This is additionally depicted in the forest plot shown in Figure 4.

**Figure 5:**
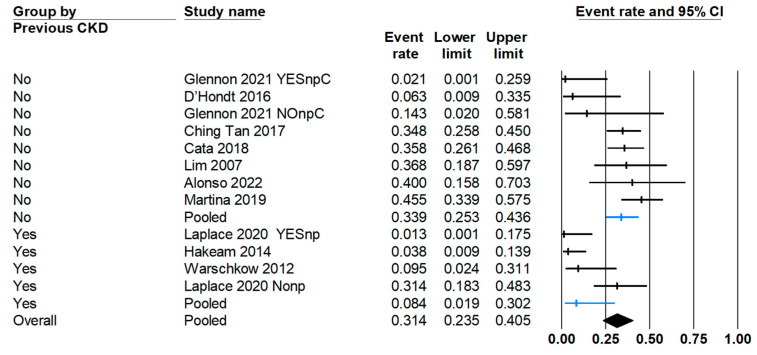
Forest plot of post cisplatin-based HIPEC AKI incidence in patients with previous CKD.

When conducting a variable effects analysis, it is observed that the presence of CKD is a protective factor against (Figure 5) the occurrence of acute renal failure following CRS and cisplatin-based HIPEC. Previous chronic kidney disease, regardless of its origin, may influence the use of cisplatin as the drug of choice for HIPEC. Some strategies employed to prevent AKI in patients with CKD include dose reduction. Alonso et al. [7] and Chen et al. [58] reduced the cisplatin dose by 25% in this subgroup of patients, although they did not specify in their analysis the number of patients with previous renal pathology included.

**Figure 6:**
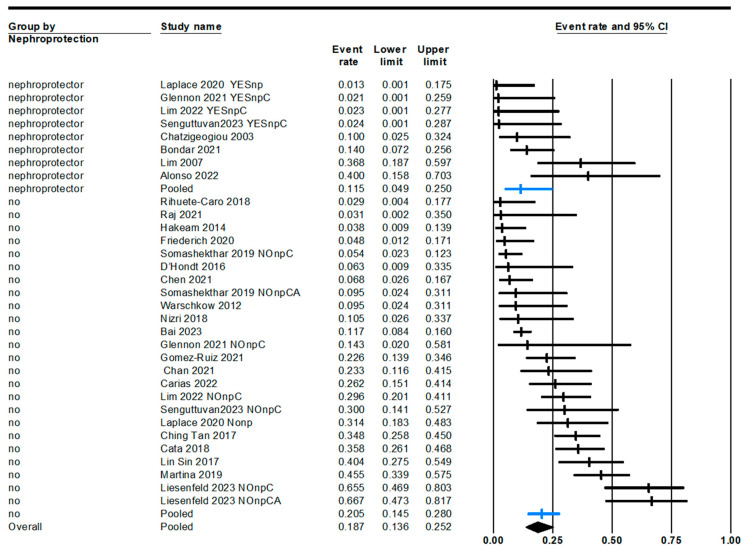
Forest plot of overall AKI incidence with or without nephroprotection (one-arm studies).

**Figure 7:**
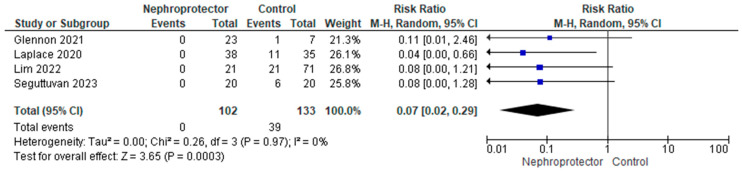
Incidence of AKI in 2-arm studies comparing the use versus non-use of nephroprotectors.

A third variable with significant impact on the incidence of renal impairment is the use of nephroprotection, regardless of whether it is ST or amifostine (Figure 6). The overall results of the use or non-use of nephroprotectors yield non-significant data. However, if we exclusively analyze studies in which a comparative design is employed, with one arm using a renal function preservation strategy and the other without it, the results differ (Figure 7). We obtain a relative risk of AKI of 7% (95% CI: 2–29%, *p* = 0.0003), favoring the use of nephroprotection. All included studies with a comparative group have a low risk of bias, but their designs vary: Laplace 2020 is an observational cohort study, Glennon 2021 is a case-control study, Senguttuvan 2023 is a non-randomized clinical trial, and Lim 2022 is a randomized clinical trial.

The remaining variables investigated do not exhibit a directly significant relationship with the incidence of AKI following cisplatin-based HIPEC administration, as shown in Table 4. Related figures are available in Supplementary Materials.

## 4. Discussion

To our knowledge, this is the first reported systematic review and meta-analysis on nephrotoxicity after complete CRS followed by cisplatin-based HIPEC. Our main goal was to determine the incidence of renal impairment (acute and chronic) associated with this treatment so that it can serve as a reference for future studies on this topic and for practice improvement or benchmarking. Additionally, we analyzed several variables that could influence the observed rates of acute and chronic renal impairment.

The unprecedented reported survival benefit by van Driel et al. in the OVIHIPEC-1 randomized clinical trial [8,10] is giving way to an increasing implementation of HIPEC with cisplatin in interval cytoreductive surgery for FIGO stage III epithelial ovarian cancer. The report of the CHIPOR trial by Classe et al. [9] (only available in abstract format at the time of writing this article), likewise showing significant survival benefits for peritoneal recurrence following induction systemic chemotherapy in this disease, adds to this phenomenon. This underscores the importance of studying short- and long-term renal toxicity following cisplatin-based HIPEC, its most concerning adverse effect. Interestingly, the dose of cisplatin employed differeed between both studies (100 mg/m^2^ in OVHIPEC-1 and 75 mg/m^2^ in CHIPOR) and neither trial specifically reported the incidence of renal impairment. For this reason, they are not included in this meta-analysis. PSOGI recently published a consensus on HIPEC regimens [140], recommending the use of the OVHIPEC-1 protocol (100 mg/m^2^ fractionated in three doses together with intravenous ST) for cisplatin-based HIPEC in epithelial ovarian cancer. Several clinical trials are underway using HIPEC with different chemotherapeutic drugs to prevent or treat PSMs from various origins, including OVHIPEC-2, which explores the use of cisplatin-based HIPEC in epithelial ovarian cancer in the upfront setting.

Reports specifically on renal impairment following cisplatin-based HIPEC are relatively scarce compared with the broad body of literature on HIPEC and are highly heterogeneous. Since awareness of this adverse effect is needed, a clear idea of its magnitude is warranted. Our meta-analysis shows an incidence of 18.7% in AKI and 7% in CKD. The observed broad range in true effects makes interpretation difficult; it becomes challenging to make a clinical decision regarding a procedure associated with such a wide-ranging serious consequence. While AKI is oftentimes manageable and transitory, a 7% incidence of chronic kidney damage following a curative-intent procedure needs to be brought to the drawing board when indicating cisplatin-based HIPEC.

Therefore, nephroprotection should be considered when using cisplatin-based HIPEC. Van Driel et al. [8] used as part of their HIPEC protocol an intravenous infusion of ST aimed at preventing renal impairment. However, CHIPOR did not use nephroprotection. ST is indicated in the treatment of cyanide poisoning. The United States Food and Drug Administration (FDA) and the European Medicines Agency (EMA) recently approved (September 2022 and March 2023, respectively) the intravenous infusion of ST to reduce hearing loss caused by cisplatin for non-metastatic solid tumors in pediatric patients. By extrapolation, off-label use of ST for renal protection is certainly an option, the same way it is used to treat calciphylaxis. Several other strategies have been reported to prevent cisplatin-based HIPEC-induced renal injury. These include the use of other intravenous nephroprotective agents (i.e., amifostine [123], cilastatin [110], forced diuresis and/or aggressive pre-hydration [141]). It is of utmost importance to keep in mind that many of the patients undergoing cisplatin-based HIPEC are likely to require additional systemic chemotherapy, making long-term organ functional preservation crucial.

Laplace et al. [6] published a prospective cohort compared with a retrospective cohort study with 66 patients diagnosed with PSMs of various origins that reflects the nephroprotective capability of ST after cisplatin-based HIPEC. The present systematic review and meta-analysis included studies using ST and amifostine, not showing differences in their capacity to prevent renal impairment. However, renal impairment was not their primary endpoint, and the assessment and categorization of renal damage was not homogeneous or standardized. In our study, the employment of nephroprotection only emerged as a significant variable impacting the incidence of renal failure when we specifically considered the studies that have two comparative arms, one using renal protection and a second arm without it. In this instance, the result differed from the overall findings, which showed no difference with the use of nephroprotection.

The most plausible explanation is that these studies were specifically designed to assess the incidence of renal failure as their primary endpoint, as an important detail that can exert a significant impact on their outcomes. All these studies include ST as nephroprotective agent and were published after the release of the initial report of van Driel et al.’s [8] study in 2018. The etiology of PSM in these studies, although varied, is mainly ovarian cancer [6,119,120,121]. Regarding the doses of cisplatin used, they range from 50 mg/m^2^ to 100 mg/m^2^, and the duration of HIPEC varies between 60 and 90 min. The rate of acute renal failure is expressed using CTCAE (Sengutuvan et al. [120] and Lim et al. [119]), while Laplace et al. [6]. use the WHO scale, and Glennon et al. [121] use KDIGO.

The renal impairment scale used to report cisplatin-based HIPEC nephrotoxicity was identified as one of the statistically significant variables impacting renal impairment incidence. To explain these findings, we searched the literature for a comparison among the different scales employed in the reports included in our meta-analysis. Although not established for CRS and HIPEC patients, it has been made for cardiac surgery patients [142], stating that RIFLE was superior to KDIGO and AKIN in terms of prognostic value for predicting mortality after isolated coronary artery bypass surgery. Additionally, Erdost et al. [143] compared these same three classifications for patients undergoing liver transplantation, another major abdominal surgery. Similarly to our findings, the results showed that AKI incidence varied depending on the scale used, and the perioperative risk factors for AKI changes with the scale as well.

These diverse renal impairment scales or classifications employed in the articles we reviewed establish different cut-off serum creatinine and/or urine output values or the need for renal replacement therapy, dialysis, in order to transition from one category to the next. They also differ in the consideration of both acute and chronic renal insufficiency. For example, when evaluating AKI using the Clavien–Dindo scale, distinguishing between grades 1 or 2 is challenging because it does not classify AKI until dialysis is required; the only difference between grade 1 and 2 is the pharmacological medical management. Exploring deeper into this comparison, AKIN 1 and 2 are analogous to CTCAE 2; meanwhile, AKIN 3 corresponds to CTCAE 3 and 4. The RIFLE scale uses cut-off points to establish Risk, Injury, and Failure stages, aligning with AKIN’s. Loss and end-stage corresponds to CKD.

**Figure 8:**
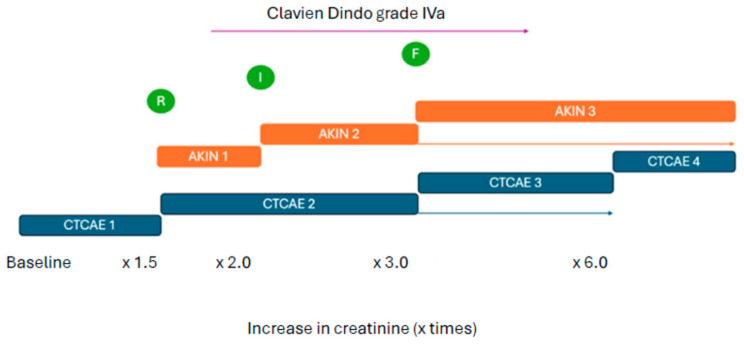
Graphical representation of acute renal impairment scales. Green: RIFLE scale; orange: AKIN scale; blue: CTCAE scale. Arrows: renal replacement therapy.

Descriptive and graphical comparisons between all scales are available in Table 5 and Figure 8, respectively. The heterogeneity in the reported data derived from this diversity makes a direct comparison of the studies considered in the present meta-analysis difficult. 

Standardization in the reporting of adverse effects within the field of surgical oncology is necessary. The use of the CTCAE scale was proposed by Younan et al. [144] as a result of an expert international consensus led by the PSOGI. However, it is true that the traditional use of the Clavien–Dindo classification in surgical services remains a practice difficult to change.

Finally, numerous clinical trials exclude patients diagnosed with any chronic organ dysfunction such as chronic kidney disease. Our study rendered an intriguing finding in that the presence of previous CKD acted as a protective factor for AKI following cisplatin-based HIPEC, with an incidence of AKI in previous CKD patients statistically significantly lower than in the non-previous CKD patient group. Several reasons might explain this finding, including the use of a cisplatin dose reduction or a more intensive monitoring of postoperative renal function in these patient groups. It is imperative to establish whether these patients could benefit from HIPEC with a presurgical organ preservation strategy. Multidisciplinary tumor conferences play a crucial role in these scenarios, where all care providers from each involved medical field must contribute, ensuring that the maximum possible number of patients can benefit from the best available treatment options.

### Strengths and Limitations

This study is the first meta-analysis and systematic review that establishes the incidence of renal impairment, acute and chronic, after cisplatin-based HIPEC for patients with PSMs. It should serve as a reference for practice improvement and future reports on this topic. The methodology adhered strictly to PRISMA recommendations.

While the findings are promising and state the need for future research, it is essential to acknowledge certain limitations. Primarily, most of the included studies include retrospective cohorts and very few are non-randomized clinical trials. Additionally, the design of the studies that met the inclusion criteria were quite diverse: sixteen cohort studies (Table 3A), two case-control studies (Table 3B), one case series (Table 3C), one randomized clinical trial (Table 3D), and seven non-randomized clinical trials. However, despite the different designs, no significant differences were found when analyzing the incidence of renal impairment based on the study design used. However, although potential biases may exist, impacting consistency, the overall risk of bias of the included studies, as evaluated by JBI Critical Appraisal Tools assessment, was low.

Additionally, the high heterogeneity observed in this research complicates result interpretation. Factors like sample size, study design, classification of renal impairment, or studies not specifically designed to evaluate adverse events as the primary endpoint may influence results. These limitations emphasize the importance of improved adverse events reporting using standardized criteria and better-designed clinical trials.

## 5. Conclusions

The results of this study reveal that the reported incidence of renal impairment following cisplatin-based HIPEC administration is highly variable because of the heterogeneity in the renal impairment scales employed. Of note, chronic renal damage can occur in 7% of patients. The use of ST and other nephroprotective drugs appears to be beneficial as a preventive strategy for acute renal injury; however, further studies are necessary to establish the optimal nephroprotective strategy in this context. It is essential to standardize the scientific communication of pharmacological toxicity to obtain higher levels of evidence that enable the standardization of results and techniques.

## Figures and Tables

**Table 1:** Extracted variables.

Study characteristics	Type of study: observational studies, either prospective or retrospective, randomized and non-randomized clinical trials.
	Unicentric or multicentric.
	Year of publication.
Population	Number of patients included.
	Number of study arms and their distribution based on HIPEC protocol and nephroprotective usage.
	Gender distribution: male or female.
	Age.
	Previous chronic renal disease (yes/no).
	Previous chemotherapy: cisplatin, other drugs, non-stated.
	Renal injury predisposing factors: ACE * inhibitors and angiotensin II receptor inhibitors, diabetes, hypertension.
	Etiology of PSM: ovarian or other.
HIPEC	HIPEC drugs: cisplatin or cisplatin + doxorubicin.
	Perfusate volume: fixed volumes or body surface area-based volume.
	Dosage: ≤50 mg/m^2^; 50–75 mg/m^2^; 75–100 mg/m^2^; ≥100 mg/m^2^.
	Duration (minutes).
	Temperature (Celsius).
	Open or closed technique.
	Nephroprotective strategy: sodium thiosulphate (ST), amifostine, no.
Kidney injury	Incidence of acute kidney injury.
	Acute kidney injury classification: RIFLE, KDIGO, AKIN, WHO, Clavien–Dindo, CTCAE.
	Incidence of chronic kidney disease.

* ACE inhibitors: angiotensin-converting enzyme (ACE) inhibitors.

**Table 2:** Characteristics of included studies.

Study	Country	Study Design	Treatment	Nephroprotector	Sample Size	Cases of Renal Impairment	Previous CKD	Tumor Etiology	Cisplatin Dose	HIPEC Duration (Minutes)	Renal Impairment Scale
Martina 2019 [122]	France	Cohort	Cisplatin	No	66	30	No	Ovary	50–100 mg/m^2^	60 min	RIFLE
Laplace 2020T [6]	France	Cohort	Cisplatin	Yes (ST)	73	11	Yes	Various	50–100 mg/m^2^	60–90 min	WHO
Lim 2007 [123]	USA	Non-randomized clinical trial	Cisplatin	Yes (amifostine)	19	7	No	Various	90 mg/m^2^ *	90 min	CTCAE
Nizri 2018 [124]	Italy	Cohort	Cisplatin and doxorubicin	No	19	2	N/A	N/A	42.5 mg/L + 15.2 mg/L **	90 min	CTCAE
Sin 2017 [125]	Singapore	Cohort	Cisplatin	No	47	19	N/A	Ovary	90 mg/m^2^	60 min	CTCAE
Somashekhar 2019T [118]	India	Cohort	Both	No	114	7	N/A	Various	75–100 mg/m^2 #^ 40 + 15 mg/m^2^ **	90 min	CTCAE
Tan 2017 [126]	Singapore	Cohort	Cisplatin	No	92	32	No	Various	40 mg/body surface	60 min	CTCAE
Warschkow 2012 [127]	Switzerland	Cohort	Cisplatin	No	21	2	Yes	Ovary	50 mg/m^2 $^	90 min	N/A
Cata 2018 [128]	USA	Cohort	Cisplatin	No	81	29	No	N/A	Not-stated	N/A	AKIN
Chan 2021 [129]	China	Non-randomized clinical trial	Cisplatin	No	30	7	N/A	Various	70 mg/m^2^, 75 mg/m^2^, 80 mg/m^2^ and 85 mg/m^2 &^	60 min	CTCAE
Friedrich 2020 [130]	Germany	Case series	Cisplatin	No	42	2	N/A	Ovary	50 mg/m^2^	90 min	CLAVIEN DINDO
Alonso 2022 [7]	Australia	Case-control	Cisplatin	Yes (ST)	10	4	No	Various	100 mg/m^2 %^	90 min	RIFLE
D’Hondt 2016 [131]	Belgium	Non-randomized clinical trial	Cisplatin	No	16	1	No	Ovary	50 mg/m^2^	60 min	CTCAE
Liesenfeld 2023 T [117]	Germany	Cohort	Both	No	56	37	N/A	Various	75 mg/m^2 %^ 50 mg/m^2^ + 15 mg/m^2^ ** 50 mg/m^2^ + 15 mg/m^2^ ** + EPIC	90 min	KDIGO
Rihuete-Caro 2018 [132]	Spain	Cohort	Cisplatin and adriamicine	No	35	1	N/A	N/A	100 mg/m^2^ + 15 mg/m^2^ **	90 min	RIFLE
Chatzigeorgiou 2003 [133]	Greece	Non-randomized clinical trial	Cisplatin	Yes (amifostine)	20	2	N/A	Ovary	50–70 mg/m^2 #^	120 min	CTCAE
Lim 2022 T [119]	South Korea	Randomized clinical trial	Cisplatin	Yes (amifostine)	92	21	N/A	Ovary	75 mg/m^2^	90 min	CTCAE
Glennon 2021 T [121]	Ireland	Case-control	Cisplatin	Yes (ST)	30	1	No	Ovary	50 mg/m^2^ 100 mg/m^2^	90 min	KDIGO
Raj 2021 [134]	India	Non-randomized clinical trial	Cisplatin	No	15	0	N/A	Ovary	75 mg/m^2^	60 min	CLAVIEN DINDO
Bondar 2021 [135]	Ukraine	Cohort	Cisplatin and adriamicine	Yes (ST)	57	8	N/A	Various	50 mg/m^2^ + 15 mg/m^2^ **	90 min	CLAVIEN DINDO
Carias 2022 [136]	Portugal	Cohort	Cisplatin	No	42	11	N/A	Various	50 mg/m^2^	90 min	KDIGO
Bai 2023 [137]	China	Cohort	Cisplatin	No	282	33	N/A	Ovary	50 to 80 mg/m^2^	60 min	KDIGO
Senguttuvan 2023 T [120]	USA	Non-randomized clinical trial	Cisplatin	Yes (ST)	40	6	N/A	Various	75 mg/m^2^	60 min	CTCAE
Chen 2021 [58]	Australia	Cohort	Cisplatin	No	59	4	N/A	Various	100 mg/m^2 %^	90 min	RIFLE
Gomez-Ruiz 2021 [138]	Spain	Cohort	Cisplatin	No	62	14	N/A	Ovary	75 mg/m^2^	60 min	RIFLE
Hakeam 2014 [139]	Saudi Arabia	Cohort	Cisplatin and adriamicine	No	53	2	Yes	Various	50 mg/m^2^ + 15 mg/m^2^ **	90 min	RIFLE

ST: sodium thiosulphate; N/A: not available; CKD: chronic kidney disease; * 1 patient received 150 mg/m^2^, 1 patient received 120 mg/m^2^; ** Cisplatin + doxorubicin; ^#^ Range dose; ^$^ 10 patients received a 30% reduction; ^&^ Dose finding trial; ^%^ 25% dose reduction if previous CKD, previous chemotherapy with 2 different drugs or previous HIPEC.

**Table 3:** (A) Risk of bias assessed by the Joanna Briggs Institute (JBI) Critical Appraisal Tools for use in cohort studies [26]. (B) Risk of bias assessed by the JBI Critical Appraisal Tools for use in case-control studies [26]. (C) Risk of bias assessed by the JBI Critical Appraisal Tools for use in case series studies [25]. (D) Risk of bias assessed by the JBI Critical Appraisal Tools for use in randomized clinical trials [24]. (E) Risk of bias assessed by the JBI Critical Appraisal Tools for use in non-randomized clinical trials [24].

(A)															
Study		JBc1	JBc2		JBc3	JBc4	JBc5	JBc6	JBc7	JBc8	JBc9	JBc10	JBc11	% Yes	Risk
Martina 2019 [122]		✓	✓		✓	✓	✓	✓	✓	✓	✓	✓	✓	100	Low
Laplace 2020 [6]		✓	✓		✓	✓	✓	✓	✓	✓	✓	✓	--	91	Low
Nizri 2018 [124]		✓	✓		✓	✓	✓	✓	✓	✓	✓	✓	✓	100	Low
Sin 2017 [125]		✓	✓		✓	✓	✓	✓	✓	✓	✓	✓	✓	100	Low
Somashekhar 2019 [118]		✓	✓		✓	✓	✓	✓	✓	✓	✓	✓	--	91	Low
Tan 2017 [126]		✓	✓		✓	✓	✓	✓	✓	✓	✓	✓	✓	100	Low
Warschkow 2012 [127]		✓	✓		✓	✓	✓	✓	U	✓	✓	✓	✓	91	Low
Cata 2018 [129]		✓	✓		✓	✓	✓	✓	✓	✓	✓	U	✓	91	Low
Liesenfeld 2023 [117]		✓	✓		✓	✓	✓	✓	✓	✓	✓	✓	✓	100	Low
Rihuete-Caro 2018 [132]		✓	✓		✓	✓	✓	✓	U	✓	✓	✓	✓	91	Low
Bondar 2021 [135]		✓	✓		✓	✓	✓	✓	✓	✓	✓	✓	✓	100	Low
Carias 2022 [136]		✓	✓		✓	✓	✓	✓	✓	✓	✓	✓	✓	100	Low
Bai 2023 [137]		✓	✓		✓	✓	✓	✓	✓	✓	U	U	✓	82	Low
Chen 2021 [58]		✓	✓		✓	✓	✓	✓	✓	✓	U	U	✓	82	Low
Gomez-Ruiz 2021 [138]		✓	✓		✓	✓	✓	✓	✓	✓	✓	✓	✓	100	Low
Hakeam 2014 [139]		✓	✓		✓	✓	✓	✓	✓	✓	U	U	✓	82	Low
**(B)**															
**Study**		**JBcc1**	**JBcc2**		**JBcc3**	**JBcc4**	**JBcc5**	**JBcc6**	**JBcc7**	**JBcc8**	**JBcc9**	**JBcc10**		**% Yes**	**Risk**
Alonso 2022 [7]		✓	✓		✓	✓	✓	--	--	✓	✓	--		70	Low
Glennon 2021 [123]		✓	✓		✓	✓	✓	✓	✓	✓	✓	--		90	Low
**(C)**															
**Study**		**JBcs1**	**JBcs2**		**JBcs3**	**JBcs4**	**JBcs5**	**JBcs6**	**JBcs7**	**JBcs8**	**JBcs9**	**JBcs10**		**% Yes**	**Risk**
Friedrich 2020 [130]		✓	✓		✓	✓	✓	✓	✓	✓	✓	✓		100	Low
**(D)**															
**Study**	**JBrct1**	**JBrct2**	**JBrct3**	**JBrct4**	**JBrct5**	**JBrct6**	**JBrct7**	**JBrct8**	**JBrct9**	**JBrct10**	**JBrct11**	**JBrct12**	**JBrct13**	**% Yes**	**Risk**
Lim 2022 [119]	✓	✓	✓	✓	--	U	✓	✓	✓	✓	✓	✓	✓	85	Low
**(E)**															
**Study**		**JBnrct1**		**JBnrct2**		**JBnrct3**	**JBnrct4**	**JBnrct5**	**JBnrct6**	**JBnrct7**	**JBnrct8**	**JBnrct9**	**% Yes**	**Risk**	
Lim 2007 [123]		✓		✓		✓	✓	✓	✓	✓	✓	--	89	Low	
Chan 2021 [129]		✓		✓		✓	--	✓	✓	✓	✓	✓	89	Low	
D’Hondt 2016 [131]		✓		✓		✓	--	--	✓	✓	✓	--	67	Moderate	
Chatzigeorgiou 2003 [133]		✓		U		U	--	✓	✓	✓	✓	✓	67	Moderate	
Raj 2021 [134]		✓		U		U	--	✓	✓	✓	✓	✓	67	Moderate	
Senguttuvan 2023 [120]		--		✓		--	✓	✓	✓	✓	✓	✓	78	Low	
Gouy 2016 [27]		✓		✓		✓	--	--	✓	✓	✓	✓	78	Low	

(A) Notes: ✓: Yes; --: No; U: Unclear; Quality items JBc1: Were the two groups similar and recruited from the same population? JBc2: Were the exposures measured similarly to assign people to both the exposed and unexposed groups? JBc3: Was the exposure measured in a valid and reliable way? JBc4: Were confounding factors identified? JBc5: Were strategies to deal with confounding factors stated? JBc6: Were the groups/participants free of the outcome at the start of the study (or at the moment of exposure)? JBc7: Were the outcomes measured in a valid and reliable way? JBc8: Was the follow-up time reported and sufficient to be long enough for outcomes to occur? JBc9: Was follow-up complete and, if not, were the reasons for loss of follow-up described and explored? JBc10: Were strategies to address incomplete follow-up utilized? JBc11: Was appropriate statistical analysis used? (B) Notes: ✓: Yes; --: No; U: Unclear; Quality items JBcc1: Were the groups comparable other than the presence of disease in cases or the absence of disease in controls? JBcc2: Were cases and controls matched appropriately? JBcc3: Were the same criteria used for the identification of cases and controls? JBcc4: Was exposure measured in a standard, valid, and reliable way? JBcc5: Was exposure measured in the same way for cases and controls? JBcc6: Were confounding factors identified? JBcc7: Were strategies to deal with confounding factors stated? JBcc8: Were outcomes assessed in a standard, valid, and reliable way for cases and controls? JBcc9: Was the exposure period of interest long enough to be meaningful? JBcc10: Was appropriate statistical analysis used? (C) Notes: ✓: Yes; --: No; U: Unclear; Quality items JBcs1: Were there clear criteria for inclusion in the case series? JBcs2: Was the condition measured in a standard, reliable way for all participants included in the case series? JBcs3: Were valid methods used for the identification of the condition for all participants included in the case series? JBcs4: Did the case series have consecutive inclusion of participants? JBcs5: Did the case series have complete inclusion of participants? JBcs6: Was there clear reporting of the demographics of the participants in the study? JBcs7: Was there clear reporting of the clinical information of the participants? JBcs8: Were the outcomes or follow-up results of cases clearly reported? JBcs9: Was there clear reporting of the presenting site(s)/clinic(s)’ demographic information? JBcs10: Was appropriate statistical analysis used? (D) Notes: ✓: Yes; --: No; U: Unclear; Quality items JBrct1: Was true randomization used for the assignment of participants to treatment groups? JBrct2: Was allocation to treatment groups concealed? JBrct3: Were treatment groups similar at the baseline? JBrct4: Were participants blind to treatment assignment? JBrct5: Were those delivering treatment blind to treatment assignment? JBrct6: Were outcomes assessors blind to treatment assignment? JBrct7: Were treatment groups treated identically other than the intervention of interest? JBrct8: Was follow-up complete and, if not, were differences between groups in terms of their follow-up adequately described and analyzed? JBrct9: Were participants analyzed in the groups to which they were randomized? JBrct10: Were outcomes measured in the same way for treatment groups? JBrct11: Were outcomes measured in a reliable way? JBrct12: Was appropriate statistical analysis used? JBrct13: Was the trial design appropriate, and any deviations from the standard RCT design (individual randomization, parallel groups) accounted for in the conduct and analysis of the trial? (E) Notes: ✓: Yes; --: No; U: Unclear; Quality items JBnrct1: Is it clear in the study what is the ‘cause’ and what is the ‘effect’ (i.e., there is no confusion about which variable comes first)? JBnrct2: Were the participants included in any comparisons similar? JBnrct3: Were the participants included in any comparisons receiving similar treatment/care, other than the exposure or intervention of interest? JBnrct4: Was there a control group? JBnrct5: Were there multiple measurements of the outcome both pre and post the intervention/exposure? JBnrct6: Was follow-up complete and, if not, were differences between groups in terms of their follow-up adequately described and analyzed? JBnrct7: Were the outcomes of participants included in any comparisons measured in the same way? JBnrct8: Were outcomes measured in a reliable way? JBnrct9: Was appropriate statistical analysis used?

**Table 4:** Main overall results and subgroup analysis of possible variables involved in AKI incidence.

Subgroup by Study Characteristics	Nº of Studies	Participants	Effect Size and 95% Interval *			Heterogeneity I^2^	*p* Value
			AKI Incidence	Lower Limit	Upper Limit		
		1473					
**Nephroprotector 1**							0.194
Yes	8	208	11.5%	4.9%	25,0%	65.0%	
No	24	1265	20.5%	14.5%	28.0%	84.8%	
**Nephroprotector 2**							0.362
Amifostine	3	60	14.0%	2.9%	46.9%	71.2%	
Sodium thiosulphate	5	148	8.8%	2.5%	27.0%	65.8%	
No nephroprotector	24	1265	20.5%	14.5%	28.0%	84.8%	
**HIPEC**							0.432
Cisplatin	26	1261	20.2%	14.6%	27,2%	81.3%	
Cisplatin and adriamicine	6	212	12.5%	3.5%	36.0%	87.7%	
**Cisplatin dose**							0.411
≤50 mg/m^2^	6	243	12.5%	6.8%	2.8%	75.3%	
>50–75 mg/m^2^	2	302	11.6%	8.4%	15.7%	0.0%	
75–100 mg/m^2^	13	519	18.8%	11.7%	28.9%	77.0%	
≥100 mg/m^2^	5	165	12.9%	4.1%	33.7%	79.5%	
**HIPEC duration**							0.465
60 min	10	650	23.2%	14.4%	35.0%	84.40%	
60–90 min	2	73	9.1%	0.3%	76.1%	83.0%	
90 min	18	649	15.2%	8.7%	25.3%	83.7%	
120 min	1	20	10.0%	2.5%	32.4%	0.0%	
**Renal impairment scale**							**0.005**
AKIN	1	81	35.8%	26.1%	46.8%	0.0%	
CLAVIEN DINDO	3	114	8.8%	3.6%	20%	32.1%	
CTCAE	15	499	17.8%	11.6%	26.3%	70.4%	
KDIGO	4	380	38.8%	13.1%	72.7%	95.3%	
RIFLE	6	285	15.4%	6.1%	33.8%	86.8%	
WHO	2	73	9.1%	0.4%	76.1%	83%	
**Tumor origin**							0.358
Ovary	13	693	15.5%	9.2%	25.1%	82.0%	
Various	16	645	21.3%	13.35	32.4%	83.8%	
**Previous kidney disease**							**0.036**
No	8	314	33.9%	25.3%	43.6%	47.9%	
Yes	4	147	8.4%	1.9%	30.2%	79.6%	
**Sex distribution (Woman %)**							0.259
≤50%	3	111	26.8%	12.9%	47.6%	56.2%	
50–75%	5	190	30.2%	11.4%	59.4%	90.0%	
>75%	21	977	16.0%	10.7%	23.1%	79.5%	
**Age distribution**							0.268
≤60 years	19	641	18.0%	12.3%	25.6%	71.3%	
>60 years	6	421	31.4%	11.9%	61.0%	93%	

**Table 5:** AKI classification scales.

Scale	Creatinine Criteria	Urine Output Criteria
RIFLE		
R (Risk)	Serum creatinine increase 1.5-fold or GFR > 25% from baseline	<0.5 mL/kg/h for 6 h
I (Injury)	Serum creatinine increase 2.0-fold or GFR > 50% from baseline	<0.5 mL/kg/h for 12 h
F (Failure)	Serum creatinine increase 3.0-fold or GFR > 75% from baseline or serum creatinine > 4.0 mg/dL with an acute increase of at least 0.5 mg/dL	Anuria for 12 h
AKIN		
1	Serum creatinine increase 0.3 mg/dL or increase to 1.5–2.0 fold from baseline	<0.5 mL/kg/h for 6 h
2	Serum creatinine increase > 2.0–3.0 fold from baseline	<0.5 mL/kg/h for 12 h
3	Serum creatinine increase > 3.0 fold from baseline or serum creatinine ≥ 4.0 mg/dL with an acute increase of at least 0.5 mg/dL or need for renal replacement therapy	<0.3 mL/kg/h for 24 h or anuria for 12 h or need for renal replacement therapy
KDIGO		
1	Serum creatinine increase 1.5–1.9 times baseline or ≥0.3 mg/dL within 48 h	<0.5 mL/kg/h for 6–12 h
2	Serum creatinine increase 2.0–2.9 times baseline	<0.5 mL/kg/h for ≥12 h
3	Serum creatinine increase 3.0 mg/dL times baseline or increase ≥ 4.0 mg/dL or initiation of renal replacement therapy	<0.3 mL/kg/h for 24 h or anuria for ≥12 h
CTCAE v. 5.0. (Acute kidney injury)		
Grade 1	-	-
Grade 2	-	-
Grade 3	Hospitalization indicated	-
Grade 4	Life-threatening consequences; dialysis indicated	-
CTCAE (Creatinine increase)		
Grade 1	Creatinine increased > upper limit normal (ULN)-1.5 × ULN	-
Grade 2	Creatinine increased > 1.5–3.0 × baseline; >1.5–3.0 × ULN	-
Grade 3	Creatinine increased > 3.0 × baseline; 3.0–6.0 × ULN	-
Grade 4	Creatinine increased > 6.0 × ULN	-
Clavien–Dindo		
Grade I	Any deviation from the normal postoperative course without the need for pharmacological treatment, or surgical, endoscopic, and radiological interventions Allowed therapeutic regimens are drugs including antiemetics, antipyretics, analgesics, and diuretics and electrolytes, and physiotherapy. This grade also includes wound infections opened at the bedside	-
Grade II	Requiring pharmacological treatment with drugs other than such allowed for grade I complications. Blood transfusions and total parenteral nutrition are also included	-
Grade III	Requiring surgical, endoscopic, or radiological intervention IIIa: Intervention not under general anesthesia IIIb: Intervention under general anesthesia	-
Grade IV	Life-threatening complication (including central nervous system complications) requiring IC/ICU management IVa: Single-organ dysfunction (including dialysis) IVb: Multiorgan dysfunction	-

## Data Availability

The original data presented in this study are openly available in Medline, Cochrane, and EMBASE.

## Supplementary Materials

The following supporting information can be downloaded at: https://www.mdpi.com/article/10.3390/jcm13133793/s1, File S1: Figures results, Search strategy and Study exclusion reasons in full-text articles assessed for eligibility.
