# Supplementary material for: Nephrotoxicity Associated with Cytoreductive Surgery Combined with Cisplatin-Based Hyperthermic Intraperitoneal Chemotherapy for Peritoneal Malignant Disease: A Systematic Review and Meta-Analysis
Source: J Clin Med. 2024 Jun 28;13(13):3793. doi: 10.3390/jcm13133793 (PMC11242517; doi:10.3390/jcm13133793)
Supplement: Supplementary file 1 [file jcm-13-03793-s001.zip › jcm-3020990-supplementary.pdf]

Journal of
Clinical Medicine

MDPI

Supplementary Material

Figure S1. HIPEC chemotherapeutic scheme

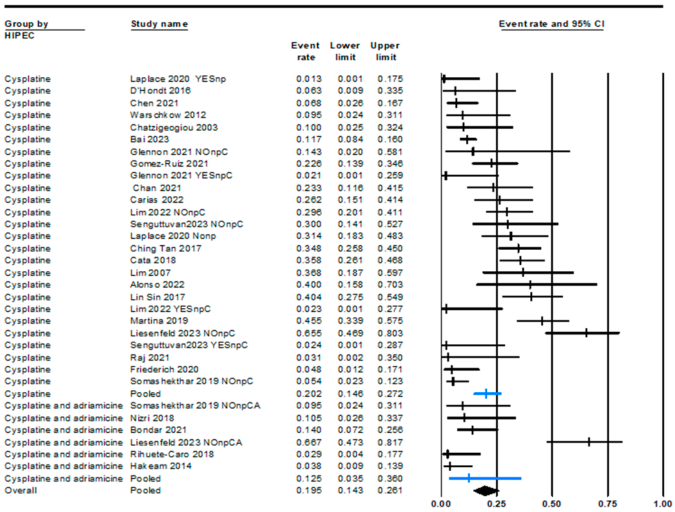

Figure S2. Cisplatin dose.

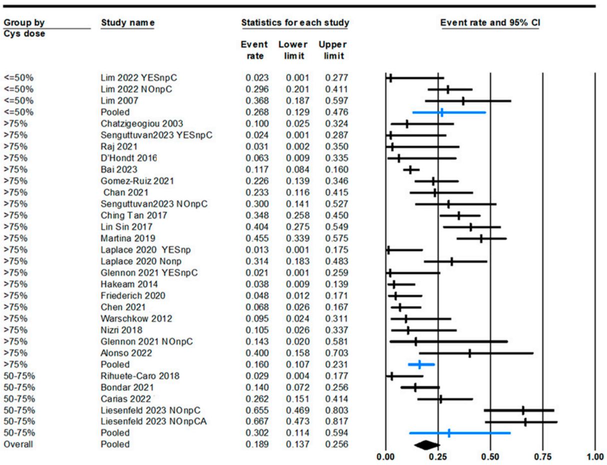

Figure S3. HIPEC duration

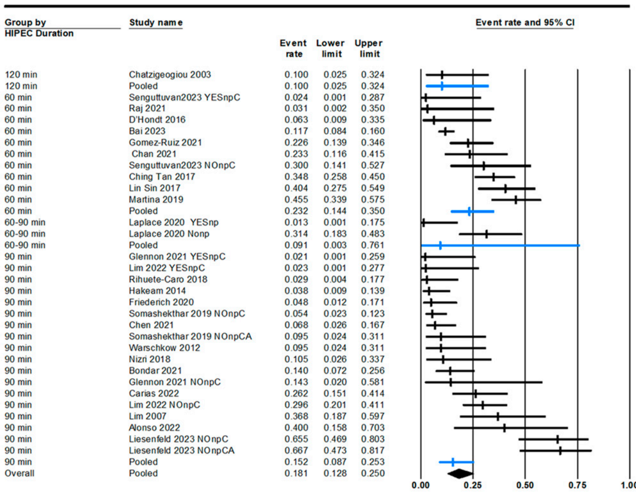

Figure S4. Etiology of peritoneal carcinomatosis

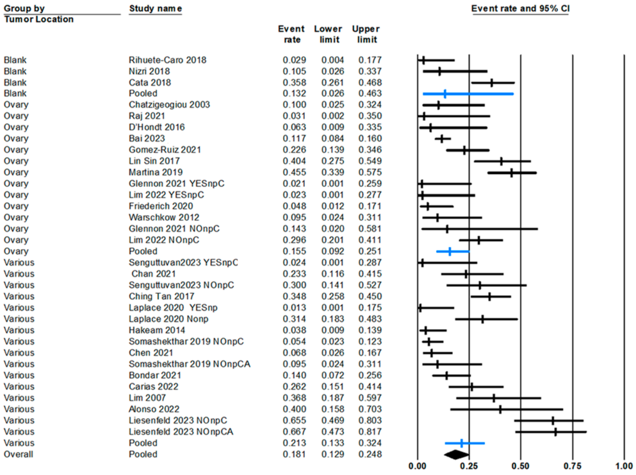

Search strategy:

Embase:

('hyperthermic intraperitoneal chemotherapy'/exp OR 'hyperthermic intraperitoneal chemotherapy' OR (hyperthermic AND intraperitoneal AND ('chemotherapy'/exp OR chemotherapy))) AND ('kidney'/exp OR kidney).

Pubmed/Medline

("hyperthermic intraperitoneal chemotherapy"(MeSH Terms) OR ("hyperthermic"(All Fields) AND "intraperitoneal"(All Fields) AND "chemotherapy"(All Fields)) OR "hyperthermic intraperitoneal chemotherapy"(All Fields) OR (("hyperthermal"(All Fields) OR "hyperthermals"(All Fields) OR "hyperthermic"(All Fields)) AND ("intraperitonal"(All Fields) OR "intraperitonally"(All Fields) OR "intraperitoneal"(All Fields) OR "intraperitoneally"(All Fields)) AND ("chemotherapy s"(All Fields) OR "drug therapy"(MeSH Terms) OR ("drug"(All Fields) AND "therapy"(All Fields)) OR "drug therapy"(All Fields) OR "chemotherapies"(All Fields) OR "drug therapy"(MeSH Subheading) OR "chemotherapy"(All Fields)))) AND ("kidney"(MeSH Terms) OR "kidney"(All Fields) OR "kidneys"(All Fields) OR "kidney s"(All Fields) OR ("renal"(All Fields) OR "renals"(All Fields)))

Translations

hyperthermic intraperitoneal chemotherapy: "hyperthermic intraperitoneal chemotherapy"(MeSH Terms) OR ("hyperthermic"(All Fields) AND "intraperitoneal"(All Fields) AND "chemotherapy"(All Fields)) OR "hyperthermic intraperitoneal chemotherapy"(All Fields)

Hyperthermic: "hyperthermal"(All Fields) OR "hyperthermals"(All Fields) OR "hyperthermic"(All Fields)

Intraperitoneal: "intraperitonal"(All Fields) OR "intraperitonally"(All Fields) OR "intraperitoneal"(All Fields) OR "intraperitoneally"(All Fields)

chemotherapy: "chemotherapy's"(All Fields) OR "drug therapy"(MeSH Terms) OR ("drug"(All Fields) AND "therapy"(All Fields)) OR "drug therapy"(All Fields) OR "chemotherapies"(All Fields) OR "drug therapy"(Subheading) OR "chemotherapy"(All Fields)

Kidney: "kidney"(MeSH Terms) OR "kidney"(All Fields) OR "kidneys"(All Fields) OR "kidney's"(All Fields)

renal: "renal"(All Fields) OR "renals"(All Fields)

Cochrane Central

((hyperthermic intraperitoneal chemotherapy) OR (Hyperthermic AND Intraperitoneal AND chemotherapy)) AND (Kidney OR renal)

Study exclusion reasons in full-text articles assessed for eligibility

Study	Reason for exclusion	Study	Reason for exclusion
Acs 2022 [28]	Wrong drug	Kluger 2010 [29]	Wrong drug
Akilli 2022 [30]	Global AKI incidence, not by HIPEC drug	Kowalsky 2023 [31]	Wrong publication type
Almerey 2018 [32]	Global AKI incidence, not by HIPEC drug	Kurreck 2022 [33]	Global AKI incidence, not by HIPEC drug
Alonso 2022 [7]	Global AKI incidence, not by HIPEC drug	La 2006 [34]	Wrong drug
Arjona-Sánchez 2016 [35]	Wrong drug	León-Póo 2023 [36]	Wrong publication type
Aronson 2023 [10]	Wrong outcome	Li 2021<br>Li 2020 [37]	Wrong drug
Ayyappan 2020 [38]	Wrong drug	Liesenfeld 2022 [39]	Wrong drug
Badgwell 2021 [40]	Wrong drug	Liesenfeld 2023 [41]	Global AKI incidence, not by HIPEC drug
Bakrin 2013 [42]	Global AKI incidence, not by HIPEC drug	Llueca 2023 [43]	Wrong outcome
Belgrano 2016 [44]	Wrong publication type	Lomnytska 2019 [45]	Wrong publication type
Benhaim 2019 [46]	Wrong drug	Loungnarath 2005 [47]	Wrong drug
Bhatt 2019 [48]	Wrong drug	Lu 2020 [49]	Wrong drug
Bouhadjari 2016 [50]	Wrong drug	Macrì 2014 [51]	Wrong drug
Cabiling 2022 [52]	Wrong publication type	Macrì 2021 [53]	Global AKI incidence, not by HIPEC drug
Chambers 2021 [54]	Global AKI incidence, not by HIPEC drug	Mehta 2018 [55]	Wrong publication type
Chen 2020 [56]	Global AKI incidence, not by HIPEC drug	Miklos 2022 [57]	Wrong publication type
Chen 2021b [58]	Wrong drug	Mitani 2022 [59]	Wrong outcome
ChiCTR2000038173 2020 [60]	Full text not available	Naffouje 2018 [61]	Global AKI incidence, not by HIPEC drug
Cioppa 2008 [62]	Wrong drug	Namendys-Silva 2013 [63]	Wrong drug
Coccolini 2015 [64]	Wrong drug	Piso 2019 [65]	Wrong outcome
Dagel 2018 [66]	Wrong population	Praiss 2022 [67]	Wrong publication type
Dellinger 2021 [68]	Wrong publication type	Račkauskas 2022 [69]	Global AKI incidence, not by HIPEC drug
Deo 2021 [70]	Global AKI incidence, not by HIPEC drug	Raft 2010 [71]	Other language
Deraco 2003 [72]	global AKI incidence, not by HIPEC drug	Randle 2015 [73]	Wrong drug
Deraco 2012 [74]	global AKI incidence, not by HIPEC drug	Roife 2020 [75]	Wrong outcome
Di Maio 2020 [76]	Wrong publication type	Roviello 2010 [77]	Wrong publication type
Di Stefano 2019 [78]	Wrong publication type	Rubio-López 2024 [79]	Wrong outcome
El-Sharkawy 2019 [80]	Wrong drug	Samel 2000 [81]	Wrong drug
Fagotti 2019 [82]	Wrong publication type	Santiago 2022 [83]	Wrong publication type
Feng 2021 [84]	Wrong drug	Somashekhar 2016 [85]	Global AKI incidence, not by HIPEC drug
Frankinet 2023 [86]	Wrong publication type	Spiegelberg 2020 [87]	Wrong drug
Giri 2017 [88]	Wrong drug	Stiles 2020 [89]	Sample size <10
Guerra-Londono 2022 [90]	Wrong outcome	Sun 2016 [91]	Global AKI incidence, not by HIPEC drug

Gupta 2021 [92]	Wrong outcome	Tan 2017 [93]	Wrong drug
Gupta 2022 [94]	Wrong outcome	Tan 2020 [95]	Global AKI incidence, not by HIPEC drug
Harkawat 2022 [96]	Wrong outcome	Tentes 2019 [97]	Wrong drug
Hendrix 2019 [98]	Wrong outcome	Vachez 2022 [99]	Wrong publication type
Hendrix 2019 [98]	Wrong drug	Vogin 2019 [100]	Wrong drug
Hsieh 2017 [101]	Wrong drug	Wallet 2016 [102]	Wrong drug
Huang 2023 [103]	Wrong publication type	Winicki 2023 [104]	Wrong outcome
Jafari 2014 [105]	HIPEC drug not stated	Wu 2022 [106]	Wrong drug
Ji 2021 [107]	Global AKI incidence, not by HIPEC drug	Ye 2018 [108]	Wrong drug
Kajdi 2014 [109]	Wrong outcome	Zaballos 2021 [110]	Wrong drug
Kepenekian 2021 [111]	Global AKI incidence, not by HIPEC drug	Zhang 2021 [112]	Wrong outcome
Khatun 2021 [113]	Wrong publication type	Zhou 2021 [114]	Wrong drug
Kianmanesh 2007 [115]	Wrong drug	Zivanovic 2015 [116]	Sample size <10
